# Refraction and Ocular Biometry of Preschool Children in Shanghai, China

**DOI:** 10.1155/2018/5205946

**Published:** 2018-03-05

**Authors:** Luoli Zhang, Xiangui He, Xiaomei Qu, Xiaofang You, Bingjie Wang, Huijing Shi, Hui Tan, Haidong Zou, Jianfeng Zhu

**Affiliations:** ^1^Eye & ENT Hospital, Fudan University, Shanghai, China; ^2^Key Laboratory of Myopia, Ministry of Health, Fudan University, Shanghai, China; ^3^Department of Preventative Ophthalmology, Shanghai Eye Disease Prevention and Treatment Center, Shanghai Eye Hospital, Shanghai, China; ^4^Department of Maternal and Child Health, School of Public Health, Key Laboratory of Public Health Safety, Ministry of Education, Fudan University, Shanghai, China

## Abstract

**Purpose:**

To investigate the refraction and ocular biometry characteristics and to examine the prevalence of refractive errors in preschool children aged 3 to 6 years in Shanghai, China.

**Methods:**

A school-based cross-sectional study was conducted in Jiading and Xuhui District, Shanghai, in 2013. We randomly selected 7 kindergartens in Jiading District and 10 kindergartens in Xuhui District, with a probability proportionate to size. The children underwent comprehensive eye examinations, including cycloplegic refraction and biometric measurements. Myopia, hyperopia, astigmatism were defined as spherical equivalent (SE) ≤ −0.50 D, SE ≥ +2.00 D, and cylindrical diopters ≤ −1.00 D.

**Results:**

The mean SE for 3- to 6-year-old children was +1.20 D (standard deviation [SD] 1.05), and the mean axial length (AL) was 22.29 mm (SD 0.73). The overall prevalence of myopia and astigmatism was 3.7% and 18.3%, respectively. No difference in prevalence of astigmatism was found across age groups. There was a statistically significant association between lower cylindrical diopters and higher spherical diopters (Spearman's correlation: −0.21, *P* < 0.001).

**Conclusion:**

Chinese children aged 3 to 6 years in the Shanghai area were mostly mildly hyperopic, with a low prevalence of myopia. Refractive astigmatism for children may be relatively stable throughout the preschool stage. Astigmatism was significantly associated with refractive error.

## 1. Introduction

The refractive status of neonates is overwhelmingly hyperopic, with a normal distribution of spherical equivalent (SE) refractive error [1–3]. Over the first year or two after birth, through a process in which the axial length (AL) of the eye elongates to match corneal power, the normal distribution of spherical equivalent refractive error narrows to be characterized by significant kurtosis [4–7]. The process of refractive development during the period through childhood and into adolescence often is described as emmetropization, but the refractive state may eventually develop towards a different refractive status other than emmetropia [8, 9]. It is necessary to investigate the refractive characteristics of the preschool stage as this period is a critical and sensitive period for visual and refractive development. The distribution of SE refraction and ocular biometric components of children aged 3 to 6 years has been studied in cities of different countries [10–15], while few studies are performed to focus on the refractive characteristics of children in Shanghai.

Many studies have found that the prevalence of myopia in school-age children and adolescents in East Asia, especially in China, is very high worldwide and higher than that in other ethnic groups [16–26]. Prevalence of myopia exceeds 60% among 12-year-old children in China after primary school, reaches nearly 80% of students aged 16 years after junior high school, and surpasses 90% in university students [27–29]. However, recent studies have shown low prevalence of myopia in Chinese preschool children. Studies conducted in Shenzhen [10], Guangzhou [11], and Xuzhou [30] city showed a low prevalence of myopia in Chinese preschool children. Another study conducted in Shanghai [31] that focused on both preschool and school-age children found a relatively low prevalence of myopia in preschool children and rapidly increasing prevalence rate after 6 years of age. But this study only showed data in one district of Shanghai.

To further understand refractive development characteristics and prevalence of refractive error in Shanghai children, this study aimed to investigate the refractive characteristics and ocular biometric parameters of preschool children in Jiading and Xuhui District of Shanghai, China, and analyze age-specific prevalence of myopia and astigmatism during the preschool stage and explore the relationship between astigmatism and refractive error.

## 2. Methods

### 2.1. Study Design

This study was part of the Elaborative Shanghai Childhood Ocular Refractive Development Study (E-SCORDS), which was supported by a Three-year Action Program of Shanghai Municipality for Strengthening the Construction of Public Health System (2011–2013; Grant number 2011-15) to establish archives of refractive status of preschool and primary school children in Shanghai and investigate myopia progression and the changes in prevalence of refractive error. The project was conducted by the Department of Eye Disease Prevention, Department of Maternal and Child Health of the School of Public Health of Fudan University, Eye and ENT Hospital of Fudan University, and Shanghai Eye Disease Prevention and Treatment Center from September 2013 to October 2014.

### 2.2. Study Population

The data of preschool children in Shanghai in the present study were from the baseline data (2013) of the E-SCORDS study. Our study was focused on the refractive status of preschool children in Jiading and Xuhui District of Shanghai. A cluster randomization based on probability proportionate to size was used. According to a previously reported myopia prevalence rate in urban China [11], a sample size of 2409 preschool children was required to attain 95% confidence intervals with a precision of 0.01, considering a cluster design effect of 1.8. Stratified cluster sampling was used. All 58 kindergartens and 84 kindergartens in Jiading and Xuhui District were divided into two levels based on the quality evaluation of the Jiading and Xuhui Education Bureau. The study design and sampling plan have also been described in previous publication [31]. A total of 2850 children from 7 kindergartens in Jiading District and 2411 children from 10 kindergartens in Xuhui District were randomly selected. A total of 4617 children participated in the examinations. Children who did not have any history of foreign ethnicity, cataract, glaucoma, or obvious retinopathy were eligible for inclusion. And in order to prevent the prevalence of astigmatism from being affected, children with normal fixation who have any history of amblyopia or strabismus were also included. With exclusion of children with a history of cataract and glaucoma (4) or foreign ethnicities (2), a total of 4611 children were included in the study. Of them, 2891 children obtained parental consent for cycloplegic refraction, and 2851 children aged 3–6 years who completed the cycloplegic refraction were analyzed in this study.

Jiading District is located in the Northwest suburban area of Shanghai, and it covers an area of 464.2 square kilometers, with a population of 1,568,000 at the end of 2015. Xuhui District is located in the southwest of downtown Shanghai, with an area of 54.8 square kilometers and a population of 1,089,100 at the end of 2015.

### 2.3. Ethics Statement

The Elaborative Shanghai Childhood Ocular Refractive Development Study (E-SCORDS) was approved by the Ethics Committee of Shanghai General Hospital. The research was conducted in accordance with the Declaration of Helsinki. The nature and possible consequences of the study were explained at each kindergarten and school. After the headmasters of the kindergartens and schools agreed to participate, the details of the examination and questionnaire were explained to the parents and guardians at a meeting prior to the examination. Written informed consent was obtained from each parent/guardian, and the children provided verbal consent on the day of the examination and survey. Examination after cycloplegia was only performed for children whose parents or guardians had given assent to their participation for all examination items. Children underwent an examination without cycloplegia, if their parents or guardians had given consent to their participation in all examination items except for cycloplegic refraction.

### 2.4. Examination

A trained team consisting of 1 ophthalmologist, 3 to 5 optometrists, 3 to 5 ophthalmic assistants, and 1 study coordinator conducted ocular examinations in 2013. A slit lamp examination and direct ophthalmoscopy were performed by an ophthalmologist to evaluate the anterior segment and the posterior segment of the eyes. Measurements of ocular biometric parameters (axial length [AL] and keratometry) were performed with an ocular biometry system (IOL Master; version 5.02, Carl Zeiss Meditec, Oberkochen, Germany). Cycloplegic refraction was performed with a desktop autorefractor (model number: KR-8800; Topcon Corporation, Tokyo, Japan). Autorefraction and corneal curvature readings of three consecutive measurements were obtained, and the average was computed automatically in each eye. Each child was reexamined until three measurements fell within 0.50 diopters (D) if any two measurements varied by >0.50 D. Cycloplegia was induced by the instillation of 1% cyclopentolate. Each child received one drop of 0.5% proparacaine hydrochloride in each eye followed by two drops of 1.0% cyclopentolate (Cyclogyl; Alcon, Fort Worth, TX, USA) with 5 minutes apart. After 30 minutes, if the pupil size was ≥6 mm and the light reflex was absent, cycloplegia was deemed adequate. Otherwise, an additional drop of proparacaine and cyclopentolate was given, and if the above standard of pupil size and light reflex had not been reached after 15 additional minutes, failure of cycloplegia was recorded. Children who lacked consent for cycloplegia also underwent autorefraction without cycloplegia.

### 2.5. Definition

After cycloplegia, spherical power and cylindrical power were measured. The cylindrical power was presented in negative notations and the spherical equivalent (SE) was calculated, which equaled the spherical power plus half of the cylindrical power. Only the data from the right eyes were included in the current study because of the high correlation between the right and left eyes (Spearman's coefficient: AL = 0.944, CR = 0.931, SE = 0.859, *P* < 0.001). Corneal radius (CR) of curvature was calculated as the mean of the longest CR and shortest CR. Myopia was defined as SE ≤ −0.50 D, emmetropia was defined as −0.50 D < SE < +0.50 D, mild hyperopia was defined as +0.50 D ≤ SE < +2.00 D, and hyperopia was defined as SE ≥ +2.00 D. Astigmatism was defined as cylindrical diopters ≤ −1.00 D. Another definition of astigmatism (cylindrical diopters ≤ −1.50 D) was also used to permit comparison with other epidemiologic studies. To classify the types of astigmatism (≤−1.00D), with-the-rule (WTR) astigmatism was defined as negative cylinder axes from 1° to 15° or from 165° to 180°, against-the-rule (ATR) astigmatism as negative cylinder axes between 75° and 105°, and oblique astigmatism as axes from 16° to 74° or from 106° to 164°. The definitions of the classification of astigmatism were chosen to facilitate comparison with other studies [11–14, 32].

### 2.6. Statistical Analysis

The comparisons of the mean values of age, SE, cylinder power, AL, CR, and the ratio of AL divided by the mean CR (AL/CR ratio) between boys and girls or districts or children with and without consent to cycloplegia were checked with the Mann–Whitney *U* test or an independent sample *t*-test. Chi-square analysis was used to compare the gender between children with and without consent to cycloplegia. Analysis of variance (ANOVA) was used to compare SE and cylinder power among age groups, and Bonferroni correction was used for post hoc analysis only when the *P* value for ANOVA was less than 0.05. Trend analysis was used to detect age differences of AL and CR. Prevalence and 95% confidence interval (CI) were calculated for different refractive categories. Chi-square analysis was used to compare the prevalence of refractive errors among age groups and between district and gender groups. Spearman's rank correlation was performed to investigate the correlations between AL and spherical power with cylindrical power. *P* values less than 0.05 were considered statistically significant. Statistical analyses were performed with SPSS 22.0 (IBM SPSS Inc., Chicago, IL, USA).

## 3. Result

### 3.1. Study Population

A total of 5261 kindergarten children were selected. Among them, 4611 children participated in the examinations at kindergartens. Written informed consent for cycloplegia was received for 2891 preschool children. Excluding those who were not suitable for, uncooperative with, or failed cycloplegia or autorefraction, a total of 2851 children aged 3 to 6 years successfully completed cycloplegic autorefraction (Table 1). The mean cylindrical diopters, CR, AL/CR ratio, and gender of the children who obtained consent to cycloplegia from parents were not statistically significantly different from those of the children without obtaining consent (cylindrical diopters: *P* = 0.248, CR: *P* = 0.281, AL/CR: *P* = 0.168, and gender: *P* = 0.862). Age and AL were statistically different between children who obtained consent and children without consent (age: *P* < 0.001 and AL: *P* = 0.021). Children who obtained the consent to cycloplegia from parents were older and had longer AL than children without consent. Considering the significant correlation between age and axial length, the axial length in children obtaining the consent was compared with that in children without obtaining consent in each age group. And no differences in AL were found between children with and without consent to cycloplegia in each age group (3-year-old age group: *P* = 0.766, 4-year-old age group: *P* = 0.118, 5-year-old age group: *P* = 0.084, and 6-year-old age group: *P* = 0.647).

The mean age was 4.86 years (standard deviation [SD], 0.82), and the mean SE for 3- to 6-year-old children was +1.20 D (SD 1.05). The mean cylindrical power was −0.55 D (SD 0.62). Detailed distributions of spherical equivalent refraction and cylindrical power by age and gender are shown in Table 2. Mean SE and cylindrical power in boys were significantly different among age groups, while the Bonferroni test for post hoc analysis found no difference between age groups. Statistically significant differences in SE were found between boys and girls across all the four age groups, with girls having more hyperopic SE than boys (data not shown).

Distributions of ocular biometry by age and gender are displayed in Table 3. The mean AL was 22.29 mm (SD 0.73). Ascending trends of mean AL were observed from 3 to 6 years of age in all the boys and girls. Significant differences in AL were found between boys and girls at all age groups, and boys had longer AL than girls at all ages. The mean CR was 7.83 mm (SD 0.27). An ascending trend of mean CR was observed from 3 to 6 years of age in boys, but this trend could not be observed in girls. Boys had significantly larger CR than girls among all age groups.

**Figure 1:**
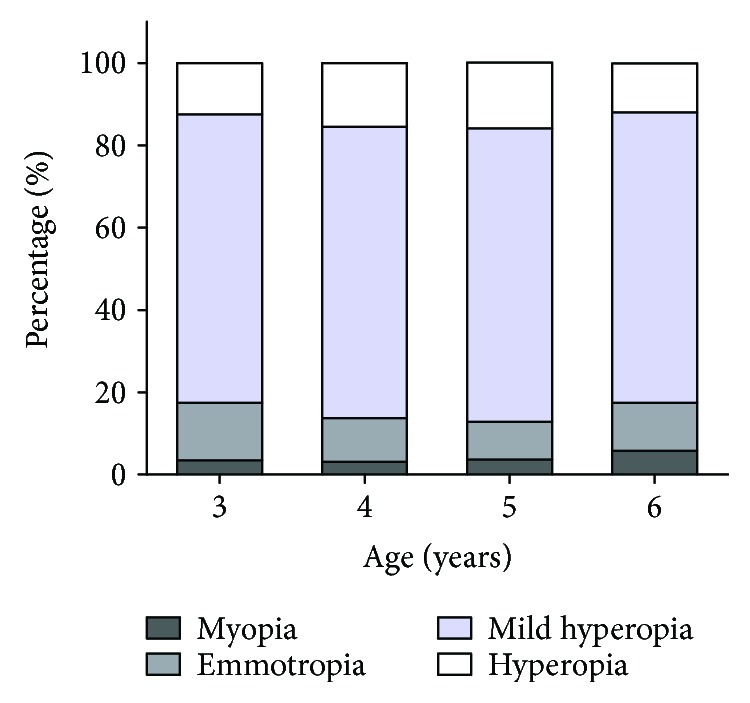
Bar graph showing age-specific distributions of the prevalence of refractive status in the right eyes.

Table 4 shows the prevalence of refractive error in different groups, and Figure 1 shows the distributions of the different refractive categories in each age group. Overall, mild hyperopia was the predominant refractive status, and myopia was not common in these 3- to 6-year-old children. The prevalence of myopia in boys was significantly different across the age groups (*P* = 0.024, chi-square analysis, data not shown), and an ascending trend of myopic prevalence was observed in boys (*P* trend = 0.027, data not shown). Children in Xuhui District demonstrated a higher prevalence of myopia than those in Jiading District (6.5% versus 2.1%; *P* < 0.001). Significant differences in the prevalence of emmetropia were found between district groups (*P* < 0.001) and gender groups (*P* = 0.006). There were no statistically significant differences in the prevalence of mild hyperopia and hyperopia across the age groups. The prevalence of mild hyperopia in Jiading district was higher than that in Xuhui District (*P* < 0.001), and girls demonstrated a higher prevalence of hyperopia than boys (*P* < 0.001).

Table 5 shows the prevalence of astigmatism (defined as cylindrical power ≤ −1.50 D or ≤−1.00 D) and axis of astigmatism in different groups. Overall, the prevalence of astigmatism for ≤−1.50 D and ≤−1.00 D was 7.4% and 18.3%, respectively. Statistically significant difference of the prevalence of astigmatism (≤−1.00D) was found between Jiading District and Xuhui District (16.9% versus 20.6%; *P* = 0.015) while no difference was found in the prevalence of astigmatism (defined as cylindrical power ≤ −1.50D) between district groups. Among the children with astigmatism (≤−1.00 D), the axis of astigmatism was with-the-rule in 81.2% and against-the-rule in 4.2%. The prevalence of WTR and oblique astigmatism was significantly different between gender groups.

Children in the astigmatism (≤−1.00 D) group had more hyperopic spherical power (1.88 D [SD: 1.54] versus 1.38 D [SD: 0.88], *P* < 0.001) and shorter axial length (22.20 mm [SD: 0.82] versus 22.32 mm [SD: 0.71], *P* = 0.002) than children in the nonastigmatism group. The prevalence of hyperopia in the astigmatism group was higher than the prevalence in the nonastigmatism group (20.9% versus 13.4%, *P* < 0.001), while the prevalence of mild hyperopia in children with astigmatism was significantly lower than that in children without astigmatism (52.6% versus 74.9%, *P* < 0.001). There was a statistically significant association between lower cylindrical power and higher spherical diopters (Spearman's correlation: −0.21, *P* < 0.001), and lower cylindrical diopters were associated with shorter axial length (Spearman's correlation: 0.102, *P* < 0.001).

## 4. Discussion

### 4.1. Refractive Parameter

In our study, the mean spherical equivalent refractive error in this group of Shanghai children aged 3 to 6 years was mildly hyperopic. This finding was similar to that of another kindergarten-based study [11] in Guangzhou, which found that the mean SE in the right eye of Chinese children across 3-, 4-, 5-, and 6-year age groups was +1.44 ± 0.76 D, +1.47 ± 0.82 D, +1.41 ± 0.82 D, and +1.33 ± 0.70 D, respectively. Studies conducted in Singapore [12]; Shenzhen, China [10]; and Xuzhou, China [30] also showed mild hyperopic status in most preschool children. The multiethnic pediatric eye disease study (MEPEDS) [13, 33], a population-based study conducted in Los Angeles County, California, showed that Hispanic, African-American, non-Hispanic white, and Asian children of preschool age were also mostly mildly hyperopic. These results show that the preschool children, regardless of ethnicity, are predominantly mildly hyperopic. Our result shows that significant differences were found in SE and cylindrical power in boys across age groups, whereas the Bonferroni test showed no differences among each age group reflecting that the mean SE and cylindrical power of children in Jiading District and Xuhui District, Shanghai, may remain stable during the preschool stage. This finding was different from that of the study conducted in Shenzhen [10] which found a descending trend of mean SE from 3 to 6 years of age but was similar to that of the study conducted in Guangzhou with no clear trends in mean SE with age over this age range [11].

### 4.2. Ocular Biometric Parameter

In our study, the mean AL and CR were longer in older children. The axial length elongation can also be observed in other investigations targeting preschool- and school-aged children [34–37]. The AL in 5-year-old and 6-year-old children from our study was 22.47 and 22.63 mm, respectively, which was similar to that in the same age children from the study conducted in Shenzhen (22.51 mm and 22.63 mm) [10] but was higher than that from one study conducted in Shandong (22.31 mm and 22.49 mm) [34]. Similar observations that CR was larger in older children were found in previous studies for school-aged children. A study [35] targeting 7- to 9-year-old children in Singapore showed that the corneal curvature radius of 9-year-old children was greater than that of 7-year-olds. Scheiman et al. [38] conducted a follow-up study observing 6- to <12-year-old children, showing that there was a slight but statistically significant flattening in corneal curvature over 14 years. However, the Shenzhen kindergarten eye study [10] showed a different result, with corneal power remaining stable during the preschool stage. And in the Anyang childhood eye study [39], it was also found that the 7-year-old children had the similar CR compared with 14-year-old children. More longitudinal studies are required to further investigate the changes in CR with age during preschool and school stages. Boys had longer AL than girls while girls had stronger corneal power than boys. Gender differences in AL and CR were consistent with those of previous studies targeting preschool children [10] and school-age children [40].

The larger CR with increasing age may to some extent explain the stable SE refraction when the axial length was elongated. Previous studies [41, 42] have reported the thinning and flattening of the crystalline lens during the preschool stage. Therefore, we hypothesize that perhaps the corneal power and lens power both flattened to compensate for the elongation of the axial length so that the SE refraction was tentatively stable during the preschool stage in our study.

### 4.3. Prevalence

In this study, the overall prevalence of myopia was 3.7% of children aged 3 to 6 years in two districts of Shanghai. Compared with the prevalence of myopia in Chinese children reported in studies conducted in Singapore [12, 43], the current study demonstrated a relatively lower prevalence of myopia (SE ≤ −0.50 D) in Shanghai, but compared with the prevalence of myopia in preschoolers reported in studies conducted in Guangzhou [11] and Shenzhen [10], the current study demonstrated a higher prevalence. In addition, the prevalence of myopia in the current study among the age groups was lower than the prevalence in Asian children reported in MEPEDS [13] but was higher than the myopia prevalence in non-Hispanic white (NHW) children (using the myopia definition of ≤−0.5 D).

The most common type of refractive error in the current study is mild hyperopia, which was similar to the findings in previous studies [13, 14, 33], and overall, the prevalence of myopia was low in this preschool population. Although higher prevalence of myopia has been reported in Chinese school-aged children and adolescents than in other ethnic groups [16, 19, 21–23], the current study and the studies targeting Chinese preschool children in other cities [10, 11, 30] have all shown relatively low prevalence of myopia, which was similar to the results from other studies [13, 14] that found low prevalence of myopia in different ethnic preschool children. This suggests that the prevalence of myopia for Chinese children is low during the preschool stage and can increase rapidly after the onset of formal schooling, indicative of a great role of environmental factors such as educational exposure for the refractive development. The patterns of rapidly increasing myopia prevalence after the age of 6 years in primary school children have been observed in Shanghai [31], Shandong [28], Guangzhou [16], Hong Kong [44, 45], and Taiwan [46, 47]. The prevalence of myopia was significantly different between the Jiading District and Xuhui District. We hypothesize that this may be due to different socioeconomic status and the different education demands between the two districts.

There was an ascending trend of the prevalence of myopia in boys while no trend was found in girls, which was similar to the finding in the Guangzhou study [11]. Conflicting results have been found, with the prevalence of myopia in the Shenzhen kindergarten eye study [10] increasing slightly with age in both boys and girls, while the MEPEDS [13] showed no age effect on the prevalence of myopia which remained relatively steady throughout the 6- to 72-month age range in both Asian and NHW children. The varied age effect on the prevalence of myopia in preschool children in different studies was probably due to environmental factors such as education and near-work-related behaviors.

The reported prevalence of astigmatism in preschool children has varied in different studies and in different ethnicities. The overall prevalence of astigmatism of 1.00 D or more (18.3%) in this study was higher than that in the Xuzhou study (8.8%) [30] and lower than that in a study conducted in Hong Kong (21.1%) [48]. Reported prevalence rates of astigmatism of 1.00 D or more in children were 4.8% in 6-year-old children in Sydney [49], 13.3% in a study conducted in Taiwan [32], and 44% in 3- to 5-year-old children in a Native American population [50]. In the current study, the prevalence of astigmatism of 1.50 D or more in the 3- and 4-year age groups was lower than the prevalence in Guangzhou, and the prevalence in the 5- and 6-year age groups was similar to that in the Guangzhou study [11]. Compared to the prevalence of astigmatism (cylinder ≤ −1.5 D) for Chinese children in Singapore [12] and African-American and Hispanic children in the MEPEDS study [51], this study showed a lower prevalence of astigmatism throughout the 36- to 72-month age range. The variation of the prevalence of astigmatism may be explained by the ethnicity, environment, testing and sample methodology, response rates, and differences in the age cohorts assessed. In our study, the WTR astigmatism was the predominant type, with 81.2% prevalence in astigmatic children, and similar findings have been reported in previous studies [12, 14, 44, 48, 52].

No difference in the prevalence of astigmatism in our study was found among age groups, and the cylindrical diopters were also stable among the 3 to 6 years of age groups. Similarly, there was also no statistically significant age effect on the prevalence of astigmatism for all preschool children in the Guangzhou study [11]. A study conducted in Baltimore, Maryland, similarly reported that the prevalence of astigmatism in African-American children was stable across age groups while a decreasing trend with age was observed in non-Hispanic white children. MEPEDS [51] reported that although an overall decrease was found in the prevalence of astigmatism throughout the 6- to 72-month age range in African-American and Hispanic children, the most rapid change occurred between 6 to 24 months in this Hispanic population. Similarly, MEPEDS [13] also showed a decreasing trend of the prevalence of astigmatism with age in younger age ranges in Asian children (<24 months) and NHW children (<30 months). It may reflect that the decreasing trend throughout the 6- to 72-month age range shown in MEPEDS was mainly due to the apparent decrease in the younger age ranges, and the prevalence of astigmatism remains stable in the 36- to 72-month age range. The study in Singapore [12] showed an increase in astigmatism prevalence with age. Our data indicated that refractive astigmatism for Shanghai children may remain relatively stable throughout the preschool stage.

**Figure 2:**
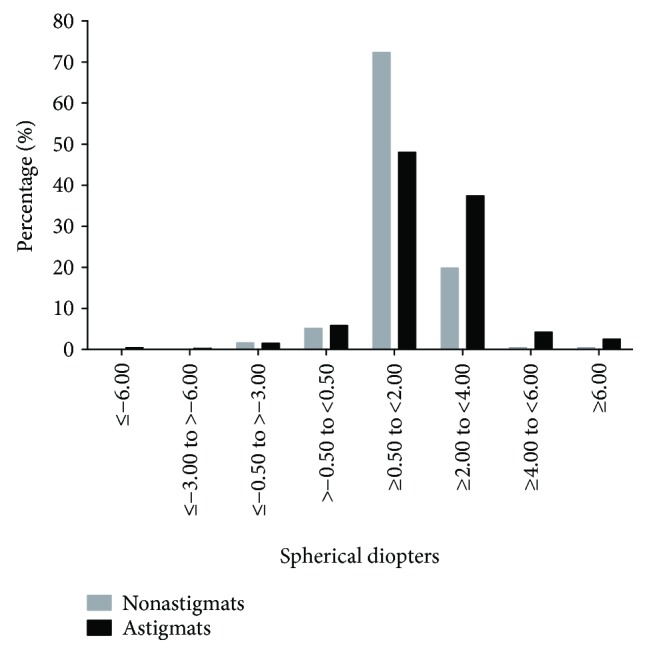
Distribution of children with astigmatism (≤−1.00 D) and without astigmatism by the classification of spherical power.

In our study, the children in the astigmatism group had greater hyperopic spherical power and shorter axial length. Children in the astigmatic group had a relatively broad distribution of spherical refractive errors, with more astigmats having hyperopic spherical diopter ≥ 2.00 D than nonastigmats, whereas in the nonastigmatic group, the data showed a tighter distribution, with most children having low hyperopic spherical diopter (Figure 2). Similar results were reported in a previous study [52]. The results that the greater mean hyperopic spherical power and the greater variability of spherical diopters in astigmats, compared with those in nonastigmats, suggest that astigmatic blur might influence emmetropization in early life. The relationship between astigmatism and refractive error is controversial. A longitudinal study [48] on preschool children in Hong Kong conducted by Fan et al. reported that at baseline, the higher the astigmatism, the more hyperopic spherical readings the children had, whereas after five years, children with higher astigmatism at baseline had greater myopic shift and longer axial length growth. The results showed in the study for Hong Kong children were not consistent with the results showed in another longitudinal study [52] targeting Tohono O'odham preschool children, demonstrating a similar myopic shift in astigmatic preschool children and nonastigmatic children over a 4- to 8-year follow-up period. The different results between the two studies may be due to the different environmental factors such as education, near-work habits, outdoor time, and the correction for refractive error over the follow-up period. More prospective studies are required to further explore the relationship between astigmatism and refractive error development.

### 4.4. Strengths and Limitations of the Study

The strength of our study lies in the randomized sampling strategy, two districts chosen which were relatively representative of the suburb and downtown area of Shanghai, objective measurements of refractive error using complete cycloplegia, and a large number of children allowing for fairly precise estimates of the prevalence of refractive errors. This could offer sound reference for understanding the refractive status of preschool children in Shanghai.

One limitation of this study was that only about two-thirds of the children obtained parental consent for cycloplegia. Due to the parental concern over side effects of cycloplegia and poor cooperation in the 3-year-old age group, the rate for cycloplegic refraction was particularly low in this group. The generally modest differences between children with and without cycloplegia would not have been expected to have a large impact. Although it is reassuring that the associations this study reported are consistent with those of some other studies involving preschool and relatively older children, the results from this study should be interpreted with caution.

## 5. Conclusion

In summary, our study provides definitive data for refractive and ocular biometry characteristics and the prevalence of refractive error in Chinese preschool children aged 3 to 6 years. On average, Shanghai preschool children in this study mostly are mildly hyperopic and have a relatively low prevalence of myopia. There was an ascending trend of the prevalence of myopia in boys, and the prevalence of astigmatism remained stable throughout the 3 to 6 years of age range. Children with higher astigmatic error had greater hyperopic spherical power and shorter axial length. Our findings aim to offer a fine reference for further longitudinal studies targeting the changes in refraction and ocular biometry and relationship between astigmatism and myopia.

## Figures and Tables

**Table 1:** Number of children examined agreeing to and completing cycloplegia.

Variable	Examined number	Consented for cycloplegia number	Completed cycloplegic refraction number
Age (Y)			
3	940	491	481
4	1660	1037	1030
5	1571	1063	1047
6	440	300	293
District			
Jiading	2746	1815	1801
Xuhui	1865	1076	1050
Gender			
Boy	2448	1532	1507
Girl	2163	1359	1344
Total	4611	2891	2851

**Table 2:** Mean refractive parameters in right eyes of 3- to 6-year-old children stratified by age and gender.

Age (years)	*N*	SE					Cylinder				
		Mean	SD	Range	Kurtosis	Skewness	Mean	SD	Range	Kurtosis	Skewness
All children	2851	1.20	1.05	22.50	21.815	0.236	−0.55	0.62	6.25	11.884	−2.69
3	481	1.12	0.96	10.63	12.892	1.854	−0.53	0.55	5.25	14.415	−2.702
4	1030	1.21	1.03	22.50	39.564	−0.875	−0.58	0.66	6.25	14.379	−3.014
5	1047	1.24	1.09	17.25	14.816	0.548	−0.54	0.61	4.50	8.4	−2.404
6	293	1.07	1.10	10.00	6.894	0.565	−0.55	0.56	3.50	5.169	−1.885
*P* value		0.033					0.41				
Boys	1507	1.10	1.05	21.38	27.014	−0.313	−0.55	0.62	6.25	13.071	−2.789
3	245	1.07	0.99	9.75	17.103	2.633	−0.49	0.49	3.50	6.726	−1.96
4	530	1.10	1.07	19.13	54.378	−3.243	−0.61	0.72	6.25	14.701	−3.157
5	561	1.18	1.04	12.50	10.937	1.552	−0.53	0.58	4.25	7.686	−2.198
6	171	0.92	1.09	9.50	5.377	−0.319	−0.53	0.56	3.25	5.467	−1.986
*P* value		0.036					0.04				
Girls	1344	1.30	1.04	18.38	16.201	0.879	−0.55	0.61	5.25	10.577	−2.581
3	236	1.19	0.94	10.13	8.465	0.953	−0.57	0.60	5.25	16.761	−3.025
4	500	1.33	0.98	10.88	16.836	2.363	−0.54	0.59	4.75	11.402	−2.591
5	486	1.32	1.14	16.88	18.569	−0.378	−0.55	0.65	4.50	8.652	−2.544
6	122	1.29	1.08	8.63	8.637	1.938	−0.59	0.57	3.50	5.114	−1.784
*P* value		0.328					0.855				

**Table 3:** Mean ocular biometry parameters (mean ± standard deviation) in the right eyes of 3- to 6-year-old children stratified by age and gender.

Characteristic	Age				*P* trend
	3	4	5	6	
Axial length (mm)					
Total	21.95 ± 0.68	22.18 ± 0.69	22.47 ± 0.71	22.63 ± 0.75	<0.001
Boys	22.21 ± 0.64	22.46 ± 0.61	22.70 ± 0.69	22.93 ± 0.68	<0.001
Girls	21.67 ± 0.61	21.87 ± 0.63	22.20 ± 0.63	22.22 ± 0.64	<0.001
*P* value	<0.001	<0.001	<0.001	<0.001	
Corneal radius (mm)					
Total	7.81 ± 0.27	7.82 ± 0.27	7.86 ± 0.26	7.87 ± 0.28	<0.001
Boys	7.88 ± 0.26	7.90 ± 0.27	7.92 ± 0.27	7.95 ± 0.27	0.007
Girls	7.73 ± 0.25	7.74 ± 0.25	7.78 ± 0.23	7.76 ± 0.25	0.099
*P* value	<0.001	<0.001	<0.001	<0.001	

**Table 4:** Prevalence of refractive errors among 3- to 6-year-old Chinese children in Shanghai.

		Myopia (SE ≤ −0.50 D)			Emmetropia (−0.50 D < SE < 0.50 D)			Mild hyperopia (0.50 D ≤ SE < 2.00 D)			Hyperopia (SE ≥ 2.00 D)		
Variable	Number	Number	%	95% confidence interval	Number	%	95% confidence interval	Number	%	95% confidence interval	Number	%	95% confidence interval
Age (years)													
3	481	17	3.5	2.1–5.2	67	13.9	11.0–17.5	337	70.1	65.7–74.2	60	12.5	9.6–15.6
4	1030	32	3.1	2.0–4.2	109	10.6	8.8–12.5	729	70.8	68.1–73.5	160	15.5	13.4–17.8
5	1047	39	3.7	2.6–4.9	95	9.1	7.4–10.9	746	71.3	68.5–74.0	167	16	13.8–18.3
6	293	17	5.8	3.4–8.5	34	11.6	8.2–15.4	207	70.6	65.2–75.8	35	11.9	8.2–15.7
*P* value		0.194			0.038			0.972			0.139		
Gender													
Boy	1507	65	4.3	3.3–5.4	184	12.2	10.5–13.7	1072	71.1	68.9–73.5	186	12.3	10.7–14.0
Girl	1344	40	3	2.1–3.9	121	9.0	7.4–10.6	947	70.5	68.2–73.0	236	17.6	15.6–19.6
*P* value		0.058			0.006			0.693			<0.001		
District													
Jiading	1801	37	2.1	1.4–2.8	160	8.9	7.7–10.2	1332	74	72.0–75.8	272	15.1	13.5–16.8
Xuhui	1050	68	6.5	5.0–8.0	145	13.8	11.7–16.0	687	65.4	62.5–68.3	150	14.3	12.3–16.5
*P* value		<0.001			<0.001			<0.001			0.554		
All	2851	105	3.7	3.0–4.4	305	10.7	9.5–11.9	2019	70.8	69.2–72.5	422	14.8	13.6–16.1

**Table 5:** Prevalence of astigmatism and axis of astigmatism among 3- to 6-year-old Chinese children in Shanghai.

		Astigmatism (≤−1.50 DC)			Astigmatism (≤−1.00 DC)			Prevalence of subtypes of astigmatism ≤ −1.00 DC								
								With-the-rule			Against-the-rule			Oblique		
Variable	Number	Number	%	95% confidence interval	Number	%	95% confidence interval	Number	%	95% confidence interval	Number	%	95% confidence interval	Number	%	95% confidence interval
Age																
3	481	28	5.8	3.7–8.1	85	17.7	14.3–21.2	60	70.6	61.2–80.0	6	7.1	2.4–12.9	19	22.4	14.1–30.6
4	1030	82	8	6.3–9.7	190	18.4	16.2–20.9	157	82.6	76.8–87.9	9	4.7	1.6–7.9	24	12.6	7.9–17.4
5	1047	78	7.4	5.9–9.1	188	18	15.6–20.3	156	83	77.7–87.8	7	3.7	1.6–6.9	25	13.3	8.5–18.1
6	293	22	7.5	4.8–10.6	58	19.8	15.4–24.2	50	86.2	77.6–94.8	0	0	N/A	8	13.8	5.2–22.4
*P* value		0.525			0.882			0.049			0.189			0.173		
Gender																
Boys	1507	110	7.3	6.0–8.6	277	18.4	16.5–20.2	213	76.9	71.9–81.9	13	4.7	2.5–7.2	51	18.4	14.1–22.7
Girls	1344	100	7.4	6.0–8.9	244	18.2	16.1–20.2	210	86.1	81.6–90.2	9	3.7	1.6–6.1	25	10.2	6.6–13.9
*P* value		0.885			0.876			0.008			0.569			0.008		
District																
Jiading	1801	122	6.8	5.6–8.0	305	16.9	15.3–18.7	249	81.6	77.0–85.9	14	4.6	2.6–7.2	42	13.8	10.2–17.7
Xuhui	1050	88	8.4	6.8–10.1	216	20.6	18.1–23.0	174	80.6	75.0–86.1	8	3.7	1.4–6.5	34	15.7	10.6–20.8
*P* value		0.113			0.015			0.755			0.62			0.53		
All	2851	210	7.4	6.4–8.4	521	18.3	16.9–19.7	423	81.2	77.7–84.5	22	4.2	2.5–6.0	76	14.6	11.7–17.5

## References

[1] Cook R. C., Glasscock R. E. (1951). Refractive and ocular findings in the newborn. *American Journal of Ophthalmology*.

[2] Pennie F. C., Wood I. C., Olsen C., White S., Charman W. N. (2001). A longitudinal study of the biometric and refractive changes in full-term infants during the first year of life. *Vision Research*.

[3] Mutti D. O., Mitchell G. L., Jones L. A. (2009). Accommodation, acuity, and their relationship to emmetropization in infants. *Optometry and Vision Science*.

[4] Mayer D. L., Hansen R. M., Moore B. D., Kim S., Fulton A. B. (2001). Cycloplegic refractions in healthy children aged 1 through 48 months. *Archives of Ophthalmology*.

[5] Mutti D. O., Mitchell G. L., Jones L. A. (2005). Axial growth and changes in lenticular and corneal power during emmetropization in infants. *Investigative Ophthalmology & Visual Science*.

[6] Brown N. P., Koretz J. F., Bron A. J. (1999). The development and maintenance of emmetropia. *Eye*.

[7] Sorsby A., Leary G. A. (1969). A longitudinal study of refraction and its components during growth. *Special Report Series (Medical Research Council (Great Britain))*.

[8] Morgan I. G., Rose K. A., Ellwein L. B., Refractive Error Study in Children Survey Group (2010). Is emmetropia the natural endpoint for human refractive development? An analysis of population-based data from the refractive error study in children (RESC). *Acta Ophthalmologica*.

[9] Siegwart J. T., Norton T. T. (2011). Perspective: how might Emmetropization and genetic factors produce myopia in normal eyes?. *Optometry and Vision Science*.

[10] Guo X., Fu M., Ding X., Morgan I. G., Zeng Y., He M. (2017). Significant axial elongation with minimal change in refraction in 3- to 6-year-old Chinese preschoolers: the Shenzhen kindergarten eye study. *Ophthalmology*.

[11] Lan W., Zhao F., Lin L. (2013). Refractive errors in 3–6 year-old Chinese children: a very low prevalence of myopia?. *PLoS One*.

[12] Dirani M., Chan Y. H., Gazzard G. (2010). Prevalence of refractive error in Singaporean Chinese children: the strabismus, amblyopia, and refractive error in young Singaporean children (STARS) study. *Investigative Ophthalmology & Visual Science*.

[13] Wen G., Tarczy-Hornoch K., McKean-Cowdin R. (2013). Prevalence of myopia, hyperopia, and astigmatism in non-Hispanic white and Asian children: multi-ethnic pediatric eye disease study. *Ophthalmology*.

[14] Giordano L., Friedman D. S., Repka M. X. (2009). Prevalence of refractive error among preschool children in an urban population: the Baltimore pediatric eye disease study. *Ophthalmology*.

[15] Hendler K., Mehravaran S., Lu X., Brown S. I., Mondino B. J., Coleman A. L. (2016). Refractive errors and amblyopia in the UCLA preschool vision program; first year results. *American Journal of Ophthalmology*.

[16] He M., Zeng J., Liu Y., Xu J., Pokharel G. P., Ellwein L. B. (2004). Refractive error and visual impairment in urban children in southern China. *Investigative Opthalmology & Visual Science*.

[17] Murthy G. V., Gupta S. K., Ellwein L. B. (2002). Refractive error in children in an urban population in New Delhi. *Investigative Ophthalmology & Visual Science*.

[18] Saw S.-M., Tong L., Chua W.-H. (2005). Incidence and progression of myopia in Singaporean school children. *Investigative Ophthalmology & Visual Science*.

[19] Dolgin E. (2015). The myopia boom. *Nature*.

[20] Lee Y. Y., Lo C. T., Sheu S. J., Yin L. T. (2015). Risk factors for and progression of myopia in young Taiwanese men. *Ophthalmic Epidemiology*.

[21] Lam C. S.-Y., Lam C.-H., Cheng S. C.-K., Chan L. Y.-L. (2012). Prevalence of myopia among Hong Kong Chinese schoolchildren: changes over two decades. *Ophthalmic and Physiological Optics*.

[22] Pan C. W., Ramamurthy D., Saw S. M. (2012). Worldwide prevalence and risk factors for myopia. *Ophthalmic and Physiological Optics*.

[23] He M., Huang W., Zheng Y., Huang L., Ellwein L. B. (2007). Refractive error and visual impairment in school children in rural southern China. *Ophthalmology*.

[24] Kleinstein R. N., Jones L. A., Hullett S. (2003). Refractive error and ethnicity in children. *Archives of Ophthalmology*.

[25] Pokharel G. P., Negrel A. D., Munoz S. R., Ellwein L. B. (2000). Refractive error study in children: results from Mechi zone, Nepal. *American Journal of Ophthalmology*.

[26] Wu L. J., You Q. S., Duan J. L. (2015). Prevalence and associated factors of myopia in high-school students in Beijing. *PLoS One*.

[27] Li S. M., Liu L. R., Li S. Y. (2013). Design, methodology and baseline data of a school-based cohort study in Central China: the Anyang childhood eye study. *Ophthalmic Epidemiology*.

[28] Wu J. F., Bi H. S., Wang S. M. (2013). Refractive error, visual acuity and causes of vision loss in children in Shandong, China. The Shandong children eye study. *PLoS One*.

[29] Sun J., Zhou J., Zhao P. (2012). High prevalence of myopia and high myopia in 5060 Chinese university students in Shanghai. *Investigative Ophthalmology & Visual Science*.

[30] Wang X., Liu D., Feng R., Zhao H., Wang Q. (2014). Refractive error among urban preschool children in Xuzhou, China. *International Journal of Clinical and Experimental Pathology*.

[31] Ma Y., Qu X., Zhu X. (2016). Age-specific prevalence of visual impairment and refractive error in children aged 3–10 years in Shanghai, China. *Investigative Ophthalmology & Visual Science*.

[32] Lai Y. H., Hsu H. T., Wang H. Z., Chang C. H., Chang S. J. (2010). Astigmatism in preschool children in Taiwan. *Journal of American Association for Pediatric Ophthalmology and Strabismus*.

[33] Multi-Ethnic Pediatric Eye Disease Study Group (2010). Prevalence of myopia and hyperopia in 6- to 72-month-old African American and Hispanic Children: the multi-ethnic pediatric eye disease study. *Ophthalmology*.

[34] Lu T. L., Wu J. F., Ye X. (2016). Axial length and associated factors in children: the Shandong children eye study. *Ophthalmologica*.

[35] Saw S. M., Carkeet A., Chia K. S., Stone R. A., Tan D. T. H. (2002). Component dependent risk factors for ocular parameters in Singapore Chinese children. *Ophthalmology*.

[36] Zadnik K., Manny R. E., Yu J. A. (2003). Ocular component data in schoolchildren as a function of age and gender. *Optometry and Vision Science*.

[37] Twelker J. D., Mitchell G. L., Messer D. H. (2009). Children’s ocular components and age, gender, and ethnicity. *Optometry and Vision Science*.

[38] Scheiman M., Gwiazda J., Zhang Q. (2016). Longitudinal changes in corneal curvature and its relationship to axial length in the correction of myopia evaluation trial (COMET) cohort. *Journal of Optometry*.

[39] Li S. M., Li S. Y., Kang M. T. (2015). Distribution of ocular biometry in 7- and 14-year-old Chinese children. *Optometry and Vision Science*.

[40] He X., Zou H., Lu L. (2015). Axial length/corneal radius ratio: association with refractive state and role on myopia detection combined with visual acuity in Chinese schoolchildren. *PLoS One*.

[41] Shih Y.-F., Chiang T.-H., Lin L. L.-K. (2009). Lens thickness changes among schoolchildren in Taiwan. *Investigative Ophthalmology & Visual Science*.

[42] Mutti D. O., Zadnik K., Fusaro R. E., Friedman N. E., Sholtz R. I., Adams A. J. (1998). Optical and structural development of the crystalline lens in childhood. *Investigative Ophthalmology & Visual Science*.

[43] Saw S. M., Chan B., Seenyen L., Yap M., Tan D., Chew S. J. (2001). Myopia in Singapore kindergarten children. *Optometry*.

[44] Fan D. S. P., Lam D. S. C., Lam R. F. (2004). Prevalence, incidence, and progression of myopia of school children in Hong Kong. *Investigative Ophthalmology & Visual Science*.

[45] Fan D. S., Cheung E. Y., Lai R. Y., Kwok A. K., Lam D. S. (2004). Myopia progression among preschool Chinese children in Hong Kong. *Annals of the Academy of Medicine, Singapore*.

[46] Lin L. L., Shih Y. F., Hsiao C. K., Chen C. J., Lee L. A., Hung P. T. (2001). Epidemiologic study of the prevalence and severity of myopia among schoolchildren in Taiwan in 2000. *Journal of the Formosan Medical Association*.

[47] Lai Y. H., Hsu H. T., Wang H. Z., Chang S. J., Wu W. C. (2009). The visual status of children ages 3 to 6 years in the vision screening program in Taiwan. *Journal of American Association for Pediatric Ophthalmology and Strabismus*.

[48] Fan D. S., Rao S. K., Cheung E. Y., Islam M., Chew S., Lam D. S. (2004). Astigmatism in Chinese preschool children: prevalence, change, and effect on refractive development. *British Journal of Ophthalmology*.

[49] Huynh S. C., Kifley A., Rose K. A., Morgan I., Heller G. Z., Mitchell P. (2006). Astigmatism and its components in 6-year-old children. *Investigative Opthalmology & Visual Science*.

[50] Dobson V., Miller J. M., Harvey E. M. (1999). Corneal and refractive astigmatism in a sample of 3- to 5-year-old children with a high prevalence of astigmatism. *Optometry and Vision Science*.

[51] Fozailoff A., Tarczy-Hornoch K., Cotter S. (2011). Prevalence of astigmatism in 6- to 72-month-old African American and Hispanic children: the multi-ethnic pediatric eye disease study. *Ophthalmology*.

[52] Dobson V., Harvey E. M., Miller J. M. (2007). Spherical equivalent refractive error in preschool children from a population with a high prevalence of astigmatism. *Optometry and Vision Science*.

